# Supplementary figures and images for: Population mobility and the development of Botswana’s generalized HIV epidemic: a network analysis
Source: medRxiv. 2023 Feb 2:2023.02.01.23285339. Preprint. [Version 1] doi: 10.1101/2023.02.01.23285339 (PMC9915826; doi:10.1101/2023.02.01.23285339)

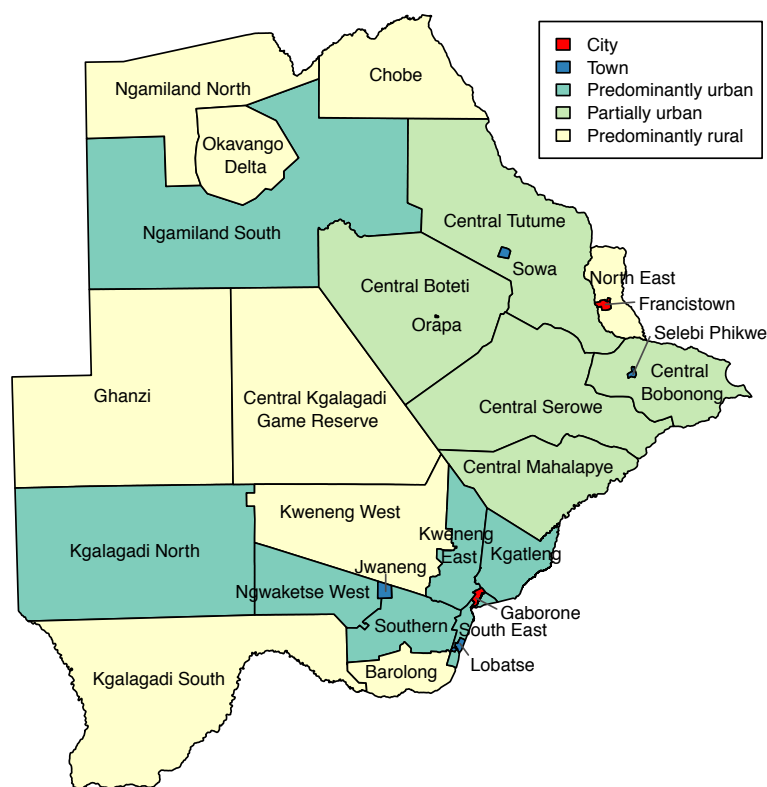

**Figure 2**—supplement 1: Map of Botswana with current district names, colored by class.

Supplement: 1 [file NIHPP2023.02.01.23285339v1-supplement-1.pdf]
